# Genetic characterization and improved genotyping of the dysferlin-deficient mouse strain *Dysf*^*tm1Kcam*^

**DOI:** 10.1186/s13395-015-0057-3

**Published:** 2015-10-13

**Authors:** Tatiana Wiktorowicz, Jochen Kinter, Kazuhiro Kobuke, Kevin P. Campbell, Michael Sinnreich

**Affiliations:** Neuromuscular Research Group, Departments of Neurology and Biomedicine, University and University Hospital Basel, Petersgraben 4, 4031 Basel, Switzerland; Howard Hughes Medical Institute, Departments of Molecular Physiology and Biophysics, Neurology, and Internal Medicine, The University of Iowa Roy J. and Lucille A. Carver College of Medicine, Iowa City, IA 52242 USA

**Keywords:** Dysferlin mouse models, Genotyping protocol, Dysferlinopathies, Targeted deletion, Knock-out

## Abstract

**Background:**

Mouse models of dysferlinopathies are valuable tools with which to investigate the pathomechanisms underlying these diseases and to test novel therapeutic strategies. One such mouse model is the *Dysf*^*tm1Kcam*^ strain, which was generated using a targeting vector to replace a 12-kb region of the dysferlin gene and which features a progressive muscular dystrophy. A prerequisite for successful animal studies using genetic mouse models is an accurate genotyping protocol. Unfortunately, the lack of robustness of currently available genotyping protocols for the *Dysf*^*tm1Kcam*^ mouse has prevented efficient colony management. Initial attempts to improve the genotyping protocol based on the published genomic structure failed. These difficulties led us to analyze the targeted locus of the dysferlin gene of the *Dysf*^*tm1Kcam*^ mouse in greater detail.

**Methods:**

In this study we resequenced and analyzed the targeted locus of the *Dysf*^*tm1Kcam*^ mouse and developed a novel PCR protocol for genotyping.

**Results:**

We found that instead of a deletion, the dysferlin locus in the *Dysf*^*tm1Kcam*^ mouse carries a targeted insertion. This genetic characterization enabled us to establish a reliable method for genotyping of the *Dysf*^*tm1Kcam*^ mouse, and thus has made efficient colony management possible.

**Conclusion:**

Our work will make the *Dysf*^*tm1Kcam*^ mouse model more attractive for animal studies of dysferlinopathies.

**Electronic supplementary material:**

The online version of this article (doi:10.1186/s13395-015-0057-3) contains supplementary material, which is available to authorized users.

## Background

Mouse models of dysferlinopathies have been developed so that the pathomechanism responsible for the dysferlinopathies can be studied, and these strains are also expected to be valuable in testing novel therapeutic strategies. Among four distinct dysferlin-deficient mouse models that have been published, two occur naturally [1, 2] and two were generated by a DNA recombination strategy [3, 1]. The first published targeted dysferlin-deficient mouse (*Dysf*^*tm1Kcam*^) was generated using a targeting vector intended to replace a 12-kb region containing the four coding exons (51–54) of the *dysf* gene; this includes exon 54, which encodes the transmembrane domain [3]. Analysis using an anti-dysferlin antibody directed against the C-terminus indicated that homozygous *Dysf*^*tm1Kcam*^ mice do not express dysferlin protein. Moreover, histological signs of muscular dystrophy are detectable by the age of 2 months, and the pathology progresses with age [3]. By the time mice are 8 months of age, the proximal muscles develop the pathological hallmarks of a muscular dystrophy (i.e., regenerating fibers, split fibers, myofiber necrosis with macrophage infiltration and muscle replacement by fatty tissue), and isolated muscle fibers from the homozygous *Dysf*^*tm1Kcam*^ mice are defective for Ca^2+^-dependent repair of the sarcolemma [3]. The *Dysf*^*tm1Kcam*^ mouse model has been used in several studies seeking to establish a better understanding of the pathomechanism of the dysferlinopathies [4–9].

We set out to establish a colony of the *Dysf*^*tm1Kcam*^ mice in order to investigate new therapeutic strategies for dysferlinopathies. However, during heterozygous breeding of these mice, we encountered difficulties in genotyping the *Dysf*^*tm1Kcam*^ progeny. When initial attempts to improve the genotyping protocol based on the published genomic structure failed, we genetically analyzed the targeted locus of the dysferlin gene of the *Dysf*^*tm1Kcam*^ mouse in greater detail. In doing so, we discovered that instead of the predicted deletion, the mice harbored a targeted insertion that accounted for the difficulties in genotyping. In this study, we resequenced and characterized the targeted locus of the *Dysf*^*tm1Kcam*^ mouse to facilitate its future use by our research groups and others. Moreover, we used smaller amplicons than those previously published, thereby reducing cycling times leading to a faster genotyping protocol.

## Methods

### Mice

The mouse strain B6.129-*Dysf*^*tm1Kcam*^/J (stock # 013149) was obtained from The Jackson Laboratory. Mice were maintained by breeding of heterozygotes in a conventional animal facility with a fixed light and dark cycle. All experiments involving animals were performed in accordance with the Swiss regulations and were approved by the veterinary commission of the Canton Basel-Stadt (Kantonales Veterinäramt BS; #2391).

### PCR amplification of genomic DNA and sequencing

**Fig. 1:**
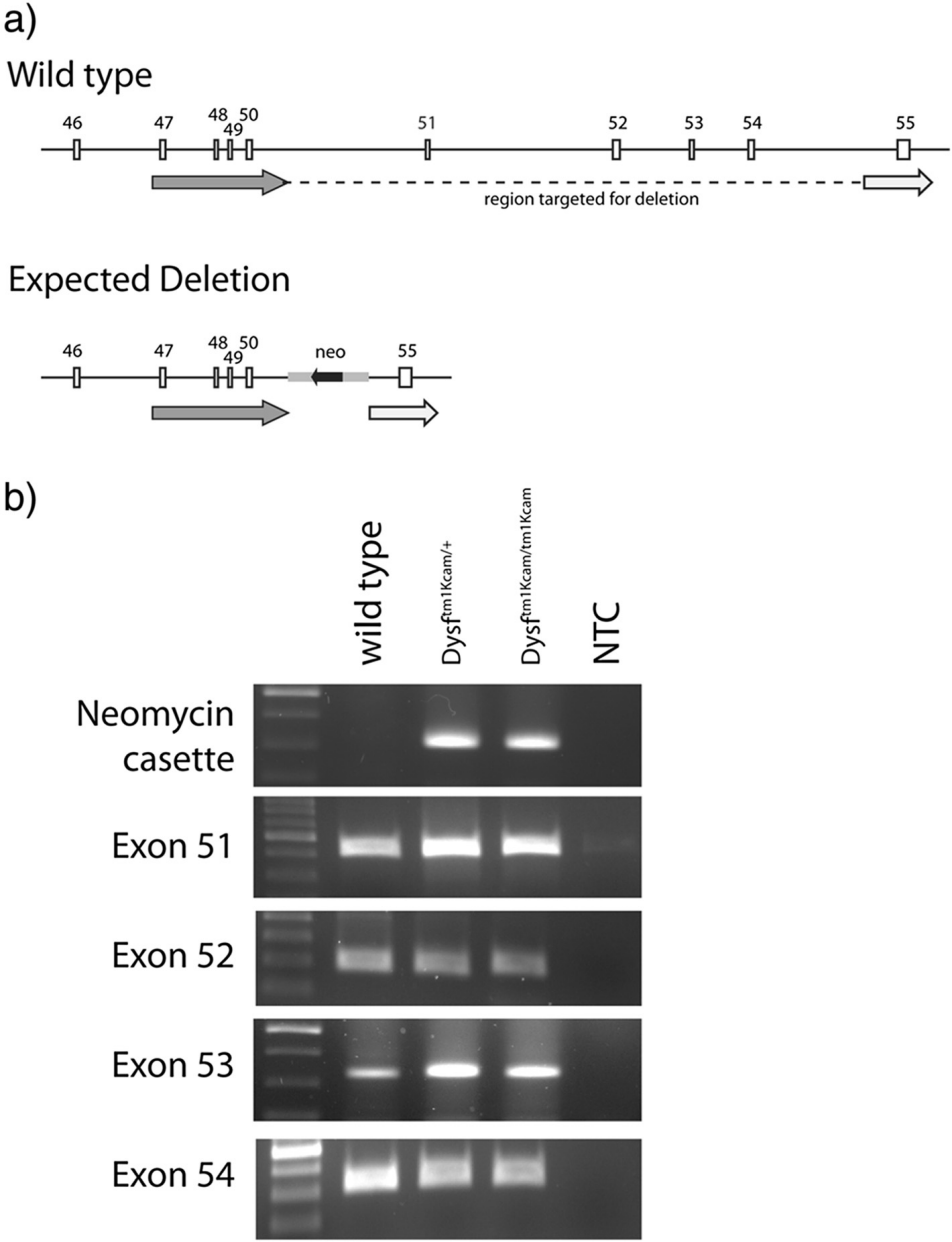
Detection of the locus targeted for deletion in homozygous *Dysf* ^*tm1Kcam*^ mice. **a** Schematic drawing of the wild-type locus and the expected deletion. The *arrows* depict the two homologous arms used for targeted deletion. **b** Results of a genotyping PCR using DNA from toe biopsies of wild type, heterozygous mutant (*Dysf* ^*tm1Kcam/+*^), and homozygous mutant (*Dysf* ^*tm1Kcam/tm1Kcam*^ *)* mice, as well as a no template control (NTC). As expected, PCR to detect the targeting vector was negative for wild type mice, and positive for heterozygous and homozygous *Dysf* ^*tm1Kcam*^ mice. However, PCR to amplify the sequence encompassing exons 51–54, which had been targeted for deletion in generating the *Dysf* ^*tm1Kcam*^ line, was positive in all mice, suggesting that homologous recombination had failed to delete the targeted region

**Fig. 2:**
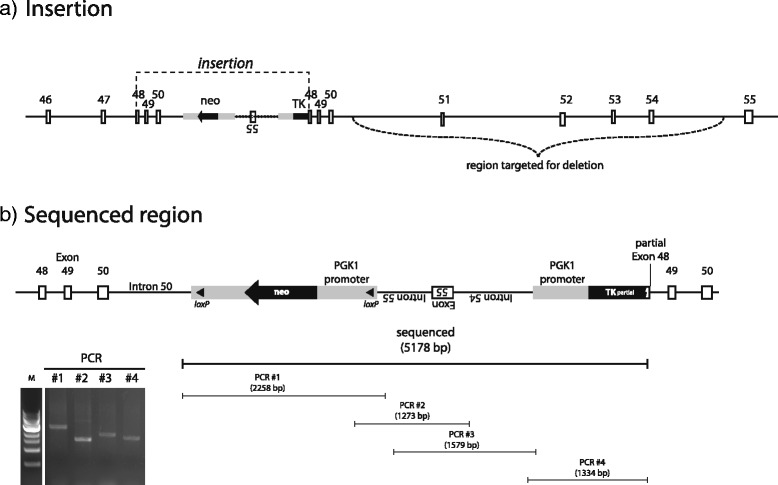
Genomic structure of the targeted dysferlin locus in the *Dysf* ^*tm1Kcam*^ mice. **a** Schematic drawing of the locus analyzed by PCR and sequencing. **b** Schematic drawing of the sequenced region, with the four PCR products generated (covering overlapping genomic regions of the targeted dysferlin locus) shown on gel and schematically. Indicated in the drawing are the neo cassette, the inverted short arm harboring exon 55, and part of the TK cassette.

Genomic DNA was extracted from liver from heterozygous B6.129-*Dysf*^*tm1Kcam*^/J mice, homozygous counterparts, and wild-type mice, by phenol chloroform extraction [10]. PCR was performed using either 5x FIREpol Master Mix (Solis BioDyne) (Fig. 1) or the Expand Long Template PCR system (Roche) (Fig. 2), according to the manufacturer’s instructions. In brief, the PCR was performed in 25 μl containing the following: 2.5 μl 10x buffer 2 (Roche), 0.25 μl 10 mM deoxynucleoside triph osphates (Sigma), 0.5 μl DNA polymerase (Roche), 1.25 μl of each specific primer (10 μM), and 2 μl DMSO. Thirty-five cycles of denaturation (92 °C, 10 s), annealing (56 °C, 30 s), and primer extension (68 °C, 1.5 min) were carried out, followed by a single extension step at the end (68 °C, 2 min), in a thermocycler (StepOnePlus, Applied Biosystems). The PCR products were sent for Sanger sequencing.

For the PCR products in Fig. 1, the following primer pairs were used:

- 5′-CAG AGA GCA AGA TCC CAG CA-3′ forward,
- 5′-TGG GGC ACA AGG ATA AGA CA-3′ reverse (exon 51–408 bp);
- 5′-ATC ACT CTG CCA CAG GCT CT-3′ forward,
- 5′- ATC TTC TTC TCG CCC TCC TC-3′ reverse (exon 52–187 bp);
- 5′-AAG CTG GAA ATG ACC TTG GA-3′ forward,
- 5′-CAC TAT CCT CCT GCC TCA GC-3′ reverse (exon 53–330 bp);
- 5′-GCG CCC CGA TAC TTC TTT-3′ forward
- 5′-AGG CTG CAG TTT CTG AGA GTT T-3′ reverse (exon 54–312 bp
- 5′-GGA GCT CAG GTG TCC AGT GT-3′ forward,
- 5′-GCC TGA AGA ACG AGA TCA GC-3′ reverse (neomycin cassette-312 bp).
- For the PCR products in Fig. 2, the following primer pairs were used:
- 5′-GGA GCT CAG GTG TCC AGT GT-3′ forward,
- 5′-CCC TGT CAC CAA GAG GCT TCT CC-3′ reverse (PCR1);
- 5′-GAA TGT GTG CGA GGC CAG AG-3′ forward,
- 5′-TGG GCT GGT AGG TGA CAA GAG-3′ reverse (PCR2);
- 5′-TGC TGC TTC TGA GGA TTA AGT CTG G-3′ forward,
- 5′-TAG AAT TCG AAC CCC TTC GGA TCC-3′ reverse (PCR3);
- 5′-GCC AGG TCA GCA AGC AGA AAG-3′ forward,
- 5′-GGT GAT GTT GAA GGG AGG TCC A-3′ reverse (PCR4).

The primers were designed based on the genomic sequence obtained from the UCSC Genome Browser (NCBI37/mm9 assembly, chr6:83958584–84166036).

### Protein extraction and Western blotting

Mouse muscles (*quadriceps*) were frozen in liquid nitrogen and pulverized (with mortar and pestle) on dry ice and then resuspended in RIPA buffer supplemented with protease inhibitor cocktail tablets (Roche). The lysates were sonicated two times for 15 s, incubated on ice for 2 h, and then centrifuged at 15,000 rpm for 20 min at 4 °C. Total protein levels in the lysates were determined using the BCA Protein Assay (Pierce). Proteins were separated on SDS polyacrylamide gel and blotted onto a nitrocellulose membrane. Membranes were blocked for 1 h in TBS containing 3 % BSA and 0.5 % Tween, and incubated for 16 h with the indicated antibody in the same buffer as above. Monoclonal antibodies against dysferlin were purchased from Abcam (Romeo I) and Vector Laboratories (NCL-Hamlet, clone Ham1/7B6), and that against α-actinin was from Sigma Aldrich. The membranes were washed with TBS containing 0.5 % Tween and incubated for 1 h with secondary antibody (HRP-conjugate goat anti-mouse IgG or HRP-conjugated goat anti-rabbit IgG) in TBS containing 3 % BSA and 0.5 % Tween (1:10,000 dilution). Membranes were washed in TBS containing 0.5 % Tween and developed using the LumiGLO Chemiluminescent substrate system (KPL).

### Improved genotyping PCR protocol

DNA was prepared by heating a mouse toe in 200 μl lysis Chelex-100 buffer (Biorad cat. # 142–1253) +5 μl Proteinase K (20 mg/ml) (Qiagen cat. # 19131) [11] at 55 °C for 120–180 min under constant shaking (800–900 rpm), and then centrifuging (14,000 rpm) the mixture for 1 min to pellet the debris, boiling for 8 min at 100 °C, and recentrifuging (14,000 rpm) for 8 min. For genotyping, 2 μl of a 1:10 dilution of the supernatant was used directly in the PCR. The genotyping protocol consists of a four-primer PCR. One primer pair identifies the wild-type allele (exon 48/intron 50) using the primer pair for_wt (5′-CAG GGG AAG CTA CAG ATG TGG ATT GA-3′) and rev_wt (5′-TTT CTG GTG GAC CCT ACT GCC ATC T-3′), producing an amplicon of 2043 bp. The second primer pair identifies the mutated allele (neomycin cassette) using primer pair neo_for (5′-AGG ATC TCC TGT CAT CTC ACC TTG CTC CTG-3′) and neo_rev (5′- AAG AAC TCG TCA AGA AGG CGA TAG AAG GCG-3′), producing an amplicon of 493 bp. Oligonucleotides were purchased from Microsynth (Balgach, CH). Both amplicons are produced in heterozygous mice. The PCR was performed in 20 μl containing the following: 4 μl Expand Long Range buffer with MgCl_2_ (Roche 04829034001), 0.5 μl of 10 mM deoxynucleoside triphosphates from the Expand Long Range dNTPack (Roche 04829034001), 0.25 μl Expand Long Range Enzyme Mix (Roche 04829034001), 0.5 μl of each wild-type specific primer (10 μM), 0.25 μl of each neo primer (10 μM), and 2 μl DMSO (Roche 04829034001). Thirty-five cycles of denaturation (92 °C, 10 s), annealing (56 °C, 15 s), and primer extension (68 °C, 105 s) were performed, followed by a single extension step (68 °C, 2 min), in a thermocycler (StepOnePlus, Applied Biosystems). The amplicons were analyzed by electrophoresis on a 2 % agarose gel, in 0.5 % TAE buffer.

## Results and discussion

Despite the recent development of an improved protocol for genotyping of the *Dysf*^*tm1Kcam*^ mouse [12], the PCR remains challenging due to the large size of the amplicons (3904 and 4846 bp). In an attempt to make the genotyping more efficient and more reliable, we switched to one of the genotyping protocols suggested by the supplier of the mice (Jackson Lab). This protocol consists of two separate PCR amplifications and is designed to detect the presence of the neo cassette (targeting vector) in the dysferlin locus and the absence of exon 53 in mutant mice. Using this protocol and the primer set specific for the mutant genotype, we found (as expected) that a ~300-bp amplicon (neomycine cassette) was produced from the genomic DNA of both heterozygous and homozygous mice, but not from that of wild-type mice (Fig. 1). The presence or absence of this amplicon was congruent with the genotypes. Unexpectedly, however, we were able to detect the presence of exons 51–54, which had been targeted for deletion (Fig. 1). This was the case for all genotypes, including what were thought to be homozygous knock-out mutants (Fig. 1). This revealed that the targeted region had not, in fact, been deleted in the *Dysf*^*tm1Kcam*^ mice.

**Fig. 3:**
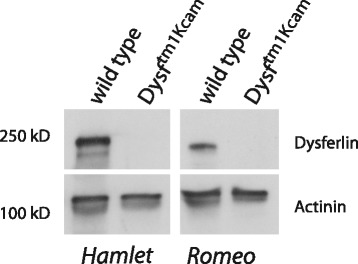
Absence of detectable dysferlin protein. Western blot of quadriceps muscle from wild-type and homozygous *Dysf* ^*tm1Kcam/tm1Kcam*^ mice using antibodies that recognize the C-terminal (NCL-Hamlet) and N-terminal (Romeo I) domains of the dysferlin protein. To demonstrate equal loading of protein, an antibody against actinin was used

We therefore performed another set of PCR reactions (Fig. 2b), followed by sequencing, to characterize the site of insertion of the neomycin cassette within the dysferlin locus of the homozygous *Dysf*^*tm1Kcam*^ mouse. Sequencing revealed that the genomic locus of the mutant mouse was indeed disrupted by the insertion of the targeting vector, but that the targeted deletion of exons 51–54 had not been achieved (Fig. 2, and Additional file 1: Supplementary material). Instead, in the *Dysf*^*tm1Kcam*^ mouse, the neomycin cassette is positioned 3′ of exon 50, followed by an inverted short arm of the targeting vector that contains exon 55 and part of the thymidine kinase cassette. Sequencing also revealed that the neomycin cassette is flanked by two LoxP sites and contains additional copies of a cloning linker; this likely led to the inverted insertion of the short arm into the targeting vector. Downstream of this vector, the genomic locus of dysferlin continues, with exon 48 followed by the remainder of the wild-type locus, including the targeted exons 51–54 (Fig. 2, and Additional file 1: Supplementary material). Importantly, despite the presence of exons 51–54, the homozygous *Dysf*^*tm1Kcam*^ mice do not synthesize full-length dysferlin (Fig. 3): neither an antibody recognizing an N-terminal epitope (Romeo I) nor one recognizing a C-terminal region of the dysferlin protein (NCL-Hamlet) detected full-length dysferlin on Western blots of muscle extracts from homozygous *Dysf*^*tm1Kcam*^ mice.

**Fig. 4:**
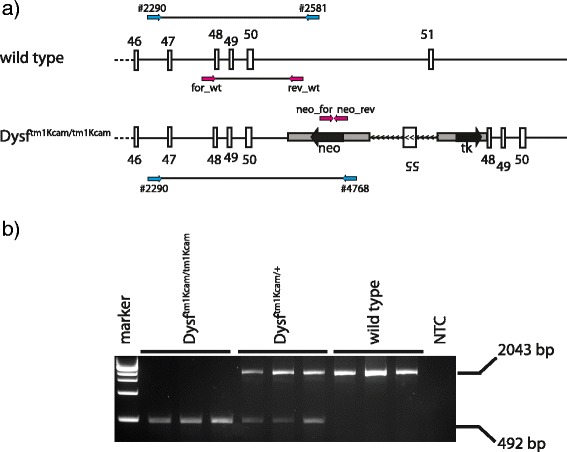
Novel genotyping PCR of *Dysf* ^*tm1Kcam*^. **a** Schematic drawing of the targeted dysferlin locus in wild type and *Dysf* ^*tm1Kcam*^ mice, with binding sites of primers used for genotyping shown in *pink*. The genotyping primers previously described by Han et al. [12] are depicted in *blue* (#2290, #2581, #4768). **b** Results of a genotyping PCR using DNA from toe biopsies of homozygous mutant (*Dysf* ^*tm1Kcam/tm1Kcam*^), heterozygous mutant (*Dysf* ^*tm1Kcam/+*^), and wild type mice, as well as a no template control (NTC)

Based on this new sequence information on the dysferlin locus in *Dysf*^*tm1Kcam*^ mice, we were able to improve the genotyping PCR by designing primers that reduce the amplicon size from ~4 kb to ~2 kb (Fig. 4). This also reduces the amplification time from the 3 h used in the most recently published protocol [12] to 1.75 h. The reductions in amplicon size and amplification time led to a robust and reliable genotyping protocol, with an error rate of about 2 %.

The finding that an insertion in the *dysf* gene rather than a deletion of several of its exons is responsible for the dysferlin deficiency in the *Dysf*^*tm1Kcam*^ mouse strain explains why attempts to improve genotyping based on published information on this locus of the Dysf^tm1Kcam^ mouse [3] have failed. We hypothesize that a homologous recombination event on the long arm led to insertion of the targeting vector, and that the reverse orientation of the short arm prevented the second recombination event that would have been necessary for the deletion of exons 51 to 54.

Despite harboring an insertion rather than a deletion on the genomic level, the Dysf^tm1Kcam^ mouse is a valid model of dysferlin deficiency, given that no full-length dysferlin protein could be detected in homozygous mice using either an N- or a C-terminally directed antibody. Homozygous *Dysf*^*tm1Kcam*^ mice show a progressive muscular dystrophy similar to that in the knock-out mouse model generated by Ho et al. [1], and the studies performed with the *Dysf*^*tm1Kcam*^ mouse model retain their validity [4, 7, 5, 6, 8, 9].

## Conclusions

We found the novel PCR protocol described here to be reliable and efficient. Due to the insertion, the size of the sequence amplified to distinguish the wild type from the mutant allele could not be reduced to below the 2 kb range. It is possible that in the past, other dysferlin-deficient mouse strains have been studied more extensively than *Dysf*^*tm1Kcam*^ because of the genotyping difficulties encountered with the latter model. The new protocol described here will allow easier characterization of this valuable dysferlinopathy mouse model, and is expected to promote its use in future studies.

## Additional file

Additional file 1: **Supplementary material Dysftm1Kcam.txt; nucleotide sequence of the sequenced region as shown in Fig.** 2b **.**

## Abbreviations

bp: base pair
DMSO: dimethyl sulfoxide
Dysf: dysferlin
kb: kilobase
PCR: polymerase chain reaction
PVDF: polyvinylidene difluoride
rpm: rounds per minute
SDS: sodium dodecyl sulfate
